# Illegal Medicine

**Published:** 1900-01

**Authors:** Nelson W. Wilson

**Affiliations:** Buffalo, N. Y.


### ILLEGAL MEDICINE.

Conducted by NELSON W. WILSON, M. D., Buffalo, N. Y.

### CONSUMPTION AND CHRISTIAN SCIENCE.

JOSEPH MORGAN died recently in Philadelphia of pulmonary tuberculosis. This in itself is not strange, but the facts surrounding the case are peculiarly interesting at this time when christian scientists are on the rampage. Morgan was fairly well-to-do. He was not under medical treatment, having been prayed over by his wife and a "healer." It was said that Mrs. Eddy was induced to come to Philadelphia and give treatments. As his end approached he suffered. His faith wavered and broke under the strain and he begged that a physician be sent for, to relieve his suffering. The best he got was a copy of the christian science *Sentinel*, which was placed in his hands, so report from Philadelphia says, and he was told to read it. Then he "passed on." His death would have been just as painful and just as certain if he had been given one of Laura Jean Libby's continued-in-our-next tales of mill-room passion.

There can be no question that Morgan's wife was sincere. Even if there was it would not be kind to raise it just at this time. Morgan was an adult capable of thinking for himself. So long as he did not want a physician all well and good. He got what he wanted—christian science, and he got it good and hard—the best there was of it, but when he did ask for a physician he should have had one, and not been deprived of the relief he could have experienced, because a smug and holy "healer," kept a blind and foolish faith-stricken wife under the power of a religious superstition.

Since the publication of this case in the newspapers, "Mother" Eddy has sent out broadcast this letter :

> Concerning the article on the death of Mr. Morgan, of Germantown, Pa., I will state I never visited Mr. Morgan's house, never attempted to heal him, never knew of the case until it was published. Have many years advertised I am not to be consulted on cases of disease, and take no patients.

The construction of this letter when compared with the published writings of the high priestess brings to mind the rumor which has been circulated that the lady who goes by the name of Mary Baker G. Eddy is a figure-head, and that the original seeress is dead and has been for some years.

---

### "PARALYSIS OF THE HEART" AND THE HEALERS.

DOWN at Rome, N. Y., Mrs. Moses M. Davis "passed on" under the ministering care of a christian scientist. Coroner Nock began an investigation.

Lizzie Moore, first-reader, and the second-reader, a man named Davidson, testified that Mrs. Davis suffered only from a delusion. They said they would confine their efforts to christian science treatment, in the case of a person who was bleeding to death. The post mortem examination showed Mrs. Davis had died of "paralysis of the heart."

---

INFECTIOUS DISEASES AND THE SCIENTISTS.

CHICAGO has epidemics of scarlet fever and measles, and the health authorities openly charge that the spread of the disease is due to failure of christian scientists to report cases, thereby preventing isolation.

---

THE BUFFALO ENQUIRER CRITICISES THE CULT.

ONE of the most common sense editorials on christian science yet printed was published recently in the *Enquirer*. It is as follows :

Christian scientists taunt medical practitioners with being "hit or miss" doctors. It is true that there are many things which the best medical skill can only guess about. It may guess right or it may guess wrong, and a human life may depend upon the guess. Medical science is still very young, as the wisest doctors know best. But medicine has a few comforts for suffering humanity which never fail.

In the report of a girl's death from cancer at South Manchester, Conn., under treatment by christian science we find this :

"Christian scientists continued to use their method of healing, but the cancer spread. Mr. Behenfield urged that a physician be called in, but the mother and brothers and sisters of the sufferer would not accede to his suggestion.

"Toward the last the girl's agonised cries so affected the neighbors that general indignation was aroused. Nothing that would deaden the pain was administered, and the patient's death was learned of with relief."

If neither medicine nor surgery could have worked a cure, medicine would, at least, have prevented the agony of this girl's last months. The merciful poppy never fails. So far as the relief of pain is concerned, medicine is an exact science. Its detractors should bear this in mind and get something equally reliable before they ask us to cut loose from it.

---

TOOTHACHE HEALERS AND THE U. OF B.

THE Rev. A. P. Moore, pastor of the Seventh Day Adventist Church of Buffalo, sent the following communication to *The Courier*, and vouched for its truthfulness :

"Mrs. Eddy, the christian science leader, found that she had a tooth that could ache. She recently went to Dr. Rae, of Concord, New Hampshire, an Adventist, and had it extracted. I consider that this spoils the theory of the so-called christian science, and proves that Mrs. Eddy is an imposter."

Edmund R. Hardy, a christian scientist, of No. 522 Ellicott Square, was asked if the statement was true.

"I don't accept it," he said. "For the last fifteen years I have heard all sorts of ridiculous reports concerning Mrs. Eddy, and I never pay any attention to them. The things that they say about her are, some of them I know personally, without foundation whatever."

"But if this were true, could you conscientiously support her?"

"Certainly. If the statement is a fact, it is in no sense an inconsistency in the christian science doctrine. We don't profess to do away with surgical operations, only the usual pain accompanying them. The last edition of the *Sentinel*, our official organ, tells of a dentist who gives christian science treatment in the place of anesthetics. Here only the other day one of the students of the University of Buffalo told me that a professor in the Dental Department said that the science treatment was coming into use in painful extractions instead of anesthetics. Mrs. Eddy might have had a tooth extracted, and it would have been entirely consistent and proper."

"And she would have suffered no pain?"

"None whatever. I can give you the testimony of a dentist who took nearly the entire nerve out of a child's tooth without the child scarcely being made aware of it."

If Mr. Hardy is correct regarding his University of Buffalo statement, the professor should be induced to give a public clinic. But on the whole Mr. Hardy's statement seems very like his defense of christian science before the committee on ordinances—very flimsy and evasive.

---

### WITCHCRAFT AT THE CENTER.

THERE is a small town in Wisconsin called Center, which must be inhabited by a most remarkable set of people. Imagine witchcraft being seriously considered in the closing days of 1899. The story as told by the newspapers is as follows:

Last August Mrs. Herman Dalke died under mysterious circumstances. Shortly afterward her children, Mary, aged 14, August, aged 8, and Antonio, 6, also died.

The peculiar circumstances surrounding the deaths caused a lot of talk and the name of John Dalke, her brother-in-law and a cripple, was mentioned unfavorably. Then a queer disease attacked stock in the neighborhood and several head of cattle died. This caused great excitement and the German settlers of the vicinity accused young Dalke of witchcraft.

The cripple instead of denying the reports claims he has supernatural powers. The neighbors threaten to lynch him. "Witch doctors'' have been appealed to and they throw out dark hints of young Dalke's powers and his possible connection with the exciting events at Center.

---

### ADULTERATION OF FOOD PRODUCTS.

SENATOR MASON, of Illinois, representing the United States Senate Committee on manufactures, has been investigating the adulteration of food products. The inquiry has been thorough and it is more than likely that results will follow, when Senator Mason makes his report to the Senate.

Prof. Russell H. Chittenden, professor of physiological chemistry at Yale University, director of the Sheffield School of Science and President of the American Physiological Society, described the action of antiseptics on the human stomach. Salicyclic acid, which the brewers have testified is used as preservative, Prof. Chittenden said retards digestion. Borax has a like effect, but he had found no deleterious effect from the use of boracic acid in small quantities.

Very few of the general food products on the market analysed by Prof. Chittenden showed the presence of antiseptics as preservatives. Prof. Chittenden said the best result would be obtained by compelling manufacturers to stamp on their products the nature and quantity of the preservatives used. He believed there should be a law prohibiting the use of coloring matter in preserving fruits and vegetables.

The witness said he had analysed all sorts of domestic liquors, including beer, and that the only adulteration he had found was an addition of water in whisky. He described beer as a hop and malt product and considered the substitution of unmalted cereals would be technically an adulteration, but not necessarily deleterious to health.

Prof. Chittenden would favor the fixing of a United States standard of purity of all food products, including liquors and the appointment of a commission of scientific experts to fix such standards.

James N. Jarvie, of Arbuckle Bros., coffee dealers and sugar refiners, told of the impurities found in the imported coffees and of the work of purifying them necessary before putting them on the market. A very small quantity, less than 1 per cent. of the total importation, is refuse coffee.

Willis G. Tucker, professor of chemistry in the Albany Medical School, and Director of the State Board of Health Laboratory, said the insufficiency of the state appropriation had prevented his doing much in the way of examining food products. Most of his time is devoted to drugs.

The law against the use of adulterants is sufficient, Dr. Tucker said, but without a large appropriation to conduct frequent analyses it is almost impossible of enforcement. For that reason, although the state has the power, it has never attempted to fix any standard of purity except in the case of vinegar, mustard and milk.

Dr. Tucker said he would have all food products carry labels, showing what antiseptics have been used in their manufacture. He favored the appointment of a national commission to fix a standard of purity of food products. Such a commission would do much to curb the popular misconception that there is a general adulteration of food. He declared his belief that there is very little such adulteration outside the imagination of the editors of various Sunday newspapers.

